# A Registry-Informed Framework for Earlier Detection of Esophageal Adenocarcinoma in Thailand: Evidence from a 16-Year Cohort Study

**DOI:** 10.3390/mps9050138

**Published:** 2026-09-21

**Authors:** Thanawat Suwatthanarak, Kullanist Thanormjit, Onchira Acharayothin, Patcharipa Chanaturakarnnon, Sripatch Sri-arporn, Tippawan Gerdsuriwong, Kanokporn Thamapala, Thapanee Saejeng, Arthit Chauykrajang, Wipanee Meesil, Tharathorn Suwatthanarak, Somsri Laiteerapong, Thammawat Parakonthun, Jirawat Swangsri, Asada Methasate, Vitoon Chinswangwatanakul, Pariyada Tanjak

**Affiliations:** 1 Siriraj Cancer Center, Faculty of Medicine Siriraj Hospital, Mahidol University, Bangkok 10700, Thailand; thanawat.suw@mahidol.edu (T.S.);; 2 Division of General Surgery, Department of Surgery, Faculty of Medicine Siriraj Hospital, Mahidol University, Bangkok 10700, Thailand; 3 Siriraj Upper Gastrointestinal Cancer Center, Department of Surgery, Faculty of Medicine Siriraj Hospital, Mahidol University, Bangkok 10700, Thailand

**Keywords:** esophageal cancer, esophageal adenocarcinoma, early detection, risk stratification, cancer registry

## Abstract

Esophageal cancer is frequently diagnosed at an advanced stage, limiting opportunities for curative treatment and contributing to poor clinical outcomes. We aimed to identify clinicopathological predictors of advanced-stage presentation and use these findings to inform a registry-informed framework for earlier detection of esophageal adenocarcinoma (AC) in Thailand. A retrospective cohort study was conducted using the Siriraj Cancer Registry. Among 1451 patients diagnosed with esophageal cancer between 2009 and 2024, 591 patients with sufficient clinical and/or pathological information for stage assignment were included. Patients were classified as having early-stage (Stage I–II) or advanced-stage (Stage III–IV) disease. Univariable and multivariable logistic regression analyses were performed to identify independent predictors of advanced-stage presentation. Among patients with sufficient information for reliable stage assignment, 77.5% presented with advanced-stage disease. AC histology was independently associated with increased odds of advanced-stage presentation compared with squamous cell carcinoma (adjusted odds ratio 4.16, 95% confidence interval 1.21–14.30; *p* = 0.024). A trend toward earlier-stage diagnosis was observed during 2017–2024 compared with 2009–2016 (adjusted odds ratio 0.66, 95% confidence interval 0.43–1.00; *p* = 0.052). These findings provide hypothesis-generating evidence to inform a risk-based approach to earlier evaluation of individuals at increased risk of esophageal AC in Thailand, which requires prospective validation.

## 1. Introduction

Esophageal cancer remains one of the most lethal malignancies worldwide, characterized by a poor prognosis and a high mortality-to-incidence ratio [1]. Globally, it ranks as the seventh most common cancer and the sixth leading cause of cancer-related mortality [2]. In Southeast Asia, particularly Thailand, esophageal cancer continues to represent a significant public health burden, with squamous cell carcinoma (SCC) historically accounting for the majority of cases [3]. However, consistent with global epidemiological trends, the incidence of esophageal adenocarcinoma (AC) has been increasing, likely reflecting changes in lifestyle and the growing prevalence of metabolic risk factors [4].

Clinical outcomes in esophageal cancer are strongly determined by the stage at diagnosis [5,6]. Patients diagnosed with localized disease (Stage I–II) have substantially better survival than those presenting with regional or distant metastases (Stage III–IV) [5,6]. Despite advances in endoscopic and imaging technologies, most patients in Thailand continue to present with advanced-stage disease, limiting opportunities for curative surgery or definitive chemoradiotherapy [3,7]. These observations underscore the need for more effective risk-based strategies to facilitate earlier diagnosis, particularly for patients at increased risk of AC.

Although previous studies have primarily focused on treatment outcomes and survival, contemporary evidence identifying clinicopathological factors associated with advanced-stage presentation in the Thai population remains limited [7,8]. Defining these risk factors may improve patient risk stratification, guide earlier endoscopic evaluation, and support the development of evidence-based early detection strategies.

In this study, we analyzed 591 patients with complete staging information from an initial cohort of 1451 patients diagnosed with esophageal cancer at Siriraj Hospital between 2009 and 2024. The Siriraj Cancer Registry is a well-established hospital-based cancer registry that adheres to the World Health Organization International Classification of Diseases for Oncology, Third Edition (ICD-O-3) standards [9,10]. Using data from this registry, we identified independent predictors of advanced-stage presentation and propose a registry-informed framework for earlier detection of esophageal AC in Thailand.

## 2. Materials and Methods

### 2.1. Study Design and Data Source

This retrospective cohort study and registry audit utilized data from the Siriraj Cancer Registry. It is a comprehensive hospital-based cancer registry at Siriraj Hospital, Mahidol University—Thailand’s largest tertiary and quaternary referral center [9]. The study protocol was approved by the Siriraj Institutional Review Board (COA no. Si 996/2025) and conducted in accordance with the Declaration of Helsinki principles.

Established in 1945, the Siriraj Cancer Center maintains a hospital-based cancer registry that is regularly audited and operated in accordance with the World Health Organization ICD-O-3 standards [10]. The registry systematically captures demographic, clinical, pathological, and treatment data for patients diagnosed or treated at the institution. Treatment variables included surgical resection, chemotherapy, and radiotherapy and were recorded as non-mutually exclusive categories. Chemotherapy and radiotherapy were not further classified according to treatment sequence or intent (e.g., neoadjuvant, adjuvant, definitive, or palliative). Treatment received outside Siriraj Hospital was included when documented in the registry. Therefore, treatment variables were analyzed descriptively and were not intended to represent stage-specific treatment strategies. Data were extracted for patients with a primary diagnosis of esophageal cancer, defined according to ICD-O-3 (code C15), diagnosed between January 2009 and December 2024 [10]. Duplicate records were identified and consolidated using unique study identification numbers to ensure data integrity.

To address incomplete staging data, cases with missing or indeterminate overall stage were reviewed using available clinical and pathological TNM information. Missing staging data were not imputed, and patients for whom an overall stage could not be reliably assigned after this review were excluded from the final analysis. The primary outcome was stage at diagnosis, categorized as early (Stage I–II) or advanced (Stage III–IV) according to the assigned American Joint Committee on Cancer (AJCC) 8th Edition stage group. For patients diagnosed under earlier AJCC editions, available clinical and pathological TNM descriptors were retrospectively reviewed and reassigned to the corresponding AJCC 8th Edition stage group rather than directly using the originally recorded stage group. When both clinical and pathological staging information were available, pTNM was prioritized, while cTNM was used when pathological staging information was unavailable. For patients who received neoadjuvant or multimodality treatment before surgery, pretreatment cTNM was used to represent the stage at presentation rather than post-treatment pathological staging. Cases for which an AJCC 8th Edition stage could not be reliably assigned were excluded from the final analysis.

### 2.2. Study Population

All patients with histologically confirmed primary epithelial esophageal malignancies (ICD-O-3 code C15) were eligible for inclusion. Patients with primary tumors of the stomach (ICD-O-3 code C16), hypopharynx, or non-epithelial esophageal malignancies (e.g., gastrointestinal stromal tumors, leiomyosarcomas, and lymphomas) were excluded to ensure a homogeneous study cohort.

For patients with multiple registry records, the entry containing the most complete clinical and pathological information was retained. Following exclusion of non-epithelial tumors and cases with insufficient information for reliable AJCC stage assignment despite review of available clinical and pathological TNM data, a final cohort of 591 patients was included in the analysis.

### 2.3. Outcome Definition

The primary outcome was overall stage at diagnosis, categorized as early (Stage I–II) or advanced (Stage III–IV) according to the AJCC 8th Edition staging system. The overall stage was determined using available clinical and/or pathological staging information. Cases with incomplete or conflicting staging data that could not be reliably classified were excluded from the final analysis.

### 2.4. Predictor Variables

The following variables were evaluated as potential predictors of advanced-stage presentation: age at diagnosis (<50 vs. ≥50 years), sex, anatomical tumor location, histological subtype, and period of diagnosis.

Tumor location was classified using ICD-O-3 topography codes into four groups: upper third (C15.0, C15.3), middle third (C15.1, C15.4), lower third including the gastroesophageal junction (C15.2, C15.5), and other/overlapping (C15.8–C15.9). The lower third served as the reference category.

Histological subtype was categorized as SCC, AC, and other epithelial malignancies (e.g., undifferentiated or small cell carcinoma), with SCC as the reference group.

The period of diagnosis was dichotomized into 2009–2016 and 2017–2024 to evaluate temporal trends, with the earlier period serving as the reference.

### 2.5. Statistical Analysis

Descriptive statistics were used to summarize patient characteristics and clinical management. Continuous variables are expressed as median (interquartile range [IQR]) and compared using the Mann–Whitney U test. Categorical variables are expressed as frequencies and percentages and compared using the chi-square test or Fisher’s exact test, as appropriate.

To assess potential selection bias due to exclusion of cases with missing staging data, baseline characteristics of included and excluded patients were compared using available demographic and clinicopathological variables.

Multivariable logistic regression was performed to identify independent predictors of advanced-stage presentation. The multivariable logistic regression model was used to identify factors independently associated with advanced-stage presentation and was not intended to serve as a clinical prediction model. Variables were selected based on clinical relevance and a trend toward association in univariable analysis (*p* < 0.20), consistent with established model-building approaches to reduce residual confounding. Adjusted odds ratios (aORs) with 95% confidence intervals (CIs) were reported.

Missing staging data were not imputed. Cases with incomplete TNM information could not be reliably assigned an overall stage and were therefore excluded from the analysis. All statistical analyses were performed using SPSS software (version 31.0; IBM Corp., Armonk, NY, USA) and Python (version 3.10). A two-sided *p* < 0.05 was considered statistically significant.

## 3. Results

### 3.1. Patient Characteristics and Cohort Selection

From an initial database of 1451 unique study identification numbers diagnosed with esophageal cancer between 2009 and 2024, a final cohort of 591 unique patients with histologically confirmed epithelial esophageal malignancies and informative staging was identified (Figure 1). The exclusion of records was primarily due to non-epithelial histology, such as leiomyosarcoma or lymphoma, or insufficient clinical and pathological data to reliably assign a TNM stage after the mapping protocol.

A total of 860 patients were excluded due to histological classification or indeterminate staging data. Comparison of baseline characteristics between included and excluded patients demonstrated no significant differences in age (mean 61.5 vs. 62.9 years, *p* = 0.081), sex distribution (male: 90.4% vs. 89.1%, *p* = 0.941), or histological subtype (*p* = 0.215) (Table S1). However, patients diagnosed in 2017–2024 were significantly more likely to have complete staging data and be included in the analysis (58.2% vs. 39.5%, *p* < 0.001).

### 3.2. Presentation and Clinical Management

As shown in Table 1, the majority of patients in the esophageal cancer cohort presented with advanced-stage disease (77.5%, n = 458), while 22.5% (n = 133) were diagnosed at an early stage (Stage I–II). Patients with early-stage disease were significantly more likely to receive radiotherapy (69.9% vs. 43.9%; *p* < 0.001) and chemotherapy (60.9% vs. 49.1%; *p* = 0.022) compared to those with advanced-stage disease. While surgical resection rates were numerically higher in the early-stage group (22.6% vs. 16.6%), the difference did not reach statistical significance (*p* = 0.147).

Anatomical distribution by tumor location showed that 11.7% of tumors were located in the upper third (cervical/upper thoracic), 35.2% in the middle third, and 15.6% in the lower third, while 37.6% were categorized as involving overlapping sites or were not otherwise specified (NOS). Notably, histological subtyping revealed that SCC remained the most common morphology (91.4%), whereas AC accounted for 7.4% of the total cohort but demonstrated a disproportionately high rate of advanced-stage presentation, comprising 9.0% of all advanced cases compared to only 2.3% of early cases (*p* = 0.011). A significant unadjusted temporal difference in stage at diagnosis was also observed, with patients diagnosed during 2017–2024 more likely to present with early-stage disease than those diagnosed during 2009–2016 (66.9% vs. 33.1%; *p* = 0.027).

### 3.3. Predictors of Advanced-Stage Presentation

The results of the univariable and multivariable logistic regression analyses identifying predictors of advanced-stage esophageal cancer are presented in Table 2 and Figure 2. In the final adjusted model, one independent predictor (AC histology) and one temporal trend were identified.

Histological subtype emerged as an independent predictor of stage at presentation. In the multivariable model, AC histology was associated with a more than four-fold increase in the odds of presenting with advanced-stage disease compared to SCC (aOR 4.16, 95% CI 1.21–14.30; *p* = 0.024). This indicates that while SCC remains more prevalent in the Thai population, patients with AC are significantly more likely to be diagnosed at Stage III–IV.

A temporal trend toward earlier-stage diagnosis was observed over the study period. Patients diagnosed in 2017–2024 had lower odds of advanced-stage presentation compared to those in 2009–2016 (aOR 0.66, 95% CI 0.43–1.00; *p* = 0.052). Although this did not reach statistical significance, it suggests a potential shift toward earlier detection at our center.

Regarding tumor location, while tumors categorized as “Other/Overlapping” showed a higher point estimate for advanced-stage presentation (aOR 1.52) compared to lower-third tumors, this association did not reach statistical significance (*p* = 0.203). Similarly, age at diagnosis (*p* = 0.577) and sex (*p* = 0.131) were not found to be independent predictors of stage at presentation after adjusting for histology and diagnosis period.

## 4. Discussion

This 16-year registry-based study provides contemporary evidence on clinicopathological factors associated with advanced-stage presentation of esophageal cancer in Thailand. It demonstrates how real-world registry data can inform a practical framework for earlier detection of esophageal AC. Our principal finding was that AC histology was independently associated with a more than fourfold increased likelihood of presenting with advanced-stage disease. Although SCC remained the predominant histological subtype in our cohort (91.4%), the substantially higher risk of late-stage diagnosis among patients with AC highlights an important opportunity for risk-based early detection in this subgroup. More broadly, while stage at diagnosis remains a major determinant of prognosis, clinicopathological heterogeneity among patients with relatively early-stage esophageal cancer may also influence clinical outcomes, highlighting the importance of refined risk stratification beyond stage alone [11].

Several factors may explain the observed association between AC and advanced-stage presentation. SCC is strongly associated with tobacco and alcohol exposure—risk factors that may prompt earlier clinical evaluation or targeted surveillance in high-risk populations [12,13]. On the other hand, AC is more commonly associated with gastroesophageal reflux disease (GERD) and Barrett’s esophagus [14,15]. In Thailand, reflux symptoms are frequently self-managed with over-the-counter medications, potentially delaying specialist referral and masking early manifestations of malignancy. In addition, AC typically arises in the distal esophagus or gastroesophageal junction (GEJ), where progressive luminal narrowing may occur later than in more proximal SCC, delaying the onset of dysphagia and clinical presentation [16]. Our findings are consistent with previous reports demonstrating that esophageal AC is commonly diagnosed at advanced stages and is associated with poor survival [17,18,19,20,21]. Collectively, these observations support a more proactive, risk-based approach that prioritizes targeted screening and timely endoscopic evaluation of individuals with chronic reflux symptoms, Barrett’s esophagus, or metabolic risk factors [22].

A notable strength of the present study is the translation of registry-derived evidence into a clinically applicable framework for earlier detection. Rather than proposing population-wide screening, we propose a risk-based framework informed by our registry findings and established external evidence. While our study identified AC histology as being associated with advanced-stage presentation, established risk factors for esophageal AC, including chronic gastroesophageal reflux symptoms, Barrett’s esophagus, and metabolic risk factors, were not captured in the present registry. Therefore, these factors are incorporated based on existing evidence rather than being directly derived from our dataset. Individuals with such established risk factors may be considered for earlier endoscopic evaluation; however, specific screening criteria and surveillance intervals require prospective evaluation in the Thai population. The regression model was designed to identify associations with advanced-stage presentation rather than to provide individualized risk prediction; therefore, the proposed framework should not be interpreted as a validated clinical prediction tool.

Beyond addressing the clinical question, this study demonstrates the value of high-quality cancer registries as platforms for translational clinical research. Well-maintained registries provide standardized real-world data that enable identification of clinically relevant risk factors, evaluation of temporal trends, and development of evidence-based strategies to improve cancer care. In the present study, the Siriraj Cancer Registry served not only as a data source but also as the foundation for developing a registry-informed framework for earlier detection of esophageal AC [9]. These findings illustrate how routinely collected registry data can be translated into practical approaches to support clinical decision-making and healthcare planning.

Although the temporal trend toward earlier-stage diagnosis during 2017–2024 did not reach statistical significance (*p* = 0.052), a higher proportion of early-stage diagnoses may reflect improvements in diagnostic pathways, including greater access to high-definition endoscopy and earlier investigation of upper gastrointestinal symptoms. Nevertheless, nearly three-quarters of patients diagnosed during the contemporary period still presented with advanced-stage disease, indicating that delayed diagnosis remains a substantial clinical challenge.

Tumor location showed an association with stage only in univariable analysis. Middle-third tumors were more frequently diagnosed at an earlier stage, possibly because lesions in this narrower segment produce dysphagia earlier, prompting medical evaluation. In contrast, overlapping or unspecified tumors were more commonly associated with advanced disease, likely reflecting extensive tumor burden that obscures the primary site at diagnosis. However, these associations were not independent predictors after multivariable adjustment.

This study has several limitations. As a retrospective registry-based analysis, information on key clinical and lifestyle variables, including body mass index, gastroesophageal reflux symptoms, Barrett’s esophagus, smoking, alcohol consumption, and other relevant risk factors, was unavailable. The absence of these variables may have resulted in residual confounding and should be considered when interpreting the observed associations.

The relatively small number of AC cases (n = 44), particularly the limited number of early-stage cases (n = 3), resulted in a wide confidence interval for the observed association (aOR 4.16, 95% CI 1.21–14.30), indicating limited precision of the effect estimate. Therefore, the AC-specific finding should be interpreted as hypothesis-generating and requires confirmation in larger, prospective cohorts.

In addition, a substantial proportion of patients were excluded because reliable stage assignment was not possible despite review of the available clinical and pathological TNM information. Although the included and excluded patients were generally comparable in age, sex, and histological subtype, patients diagnosed during 2017–2024 were more likely to have complete staging information and to be included in the analysis. This difference may have introduced selection bias and may partly influence the observed temporal trend toward earlier-stage diagnosis in the more recent period. Therefore, the temporal findings should be interpreted cautiously, and residual selection bias related to unmeasured differences between included and excluded patients cannot be excluded. Future studies with more complete longitudinal staging data may incorporate sensitivity analyses to further assess the robustness of temporal trends.

Finally, the proposed framework was derived from retrospective observational data and has not yet been prospectively validated. Future multicenter studies are still needed to evaluate its clinical utility, diagnostic performance, and cost-effectiveness.

Despite these limitations, the large contemporary cohort, standardized ICD-O-based classification, and comprehensive cancer registry provide a robust foundation for identifying patients at increased risk of advanced-stage disease. Our findings support a registry-informed framework that integrates clinicopathological risk stratification with earlier endoscopic evaluation to facilitate timely detection of esophageal AC in Thailand.

## 5. Conclusions

While SCC remains the predominant histological subtype of esophageal cancer in Thailand, our findings suggest that AC may represent a subgroup at increased risk of advanced-stage presentation. These findings provide hypothesis-generating evidence to inform risk-based approaches to earlier evaluation, rather than a validated screening strategy. Prospective multicenter studies are needed to confirm the AC-specific association and evaluate the clinical utility of the proposed approach.

## Figures and Tables

**Figure 1:**
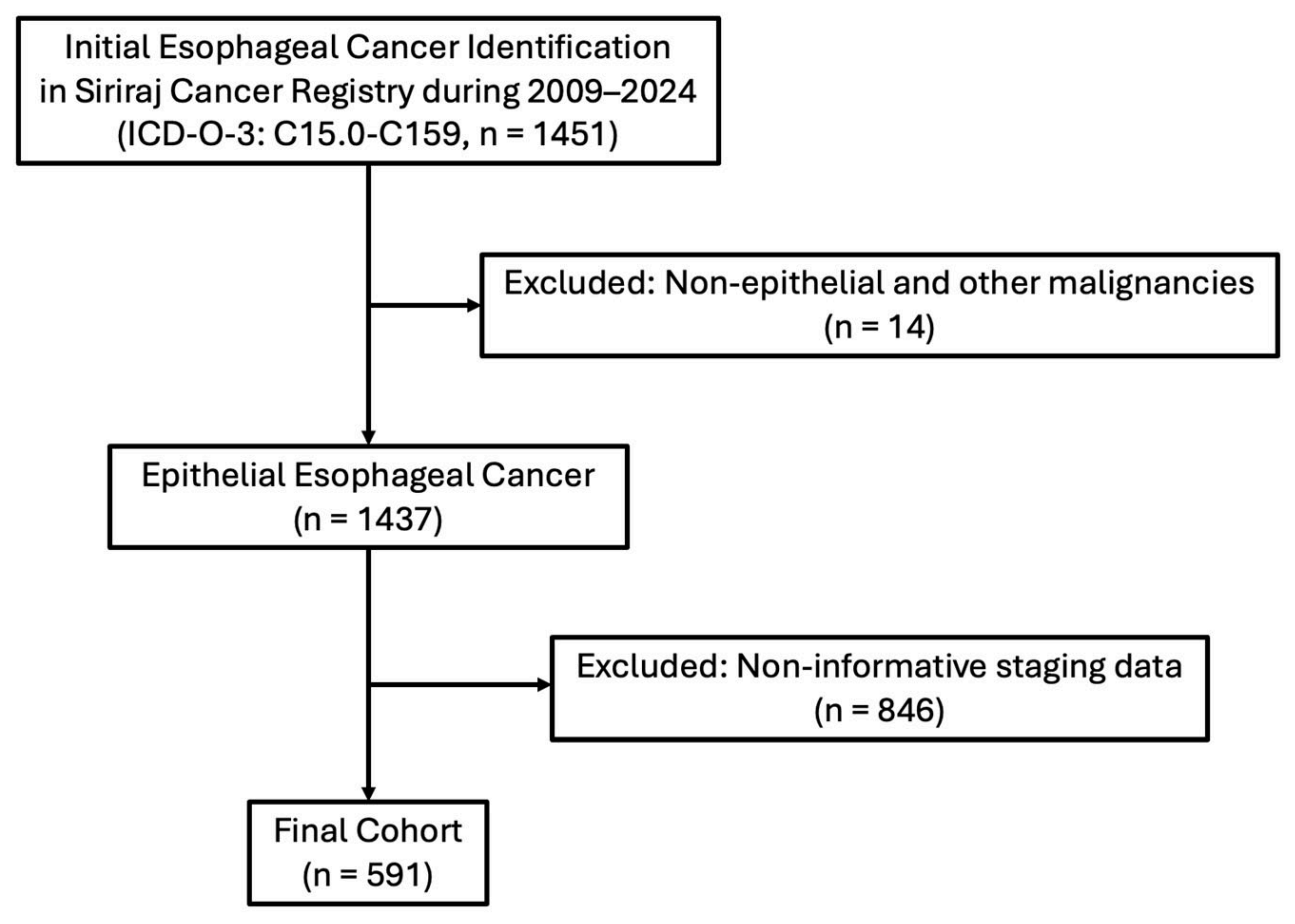
Schematic representation of the study population selection process. From an initial pool of 1451 esophageal cancer records at a quaternary referral center, exclusions were made based on histological classification and the availability of definitive staging data. The final analysis was performed on a cohort of 591 patients, categorized into early-stage and advanced-stage groups for comparative evaluation.

**Figure 2:**
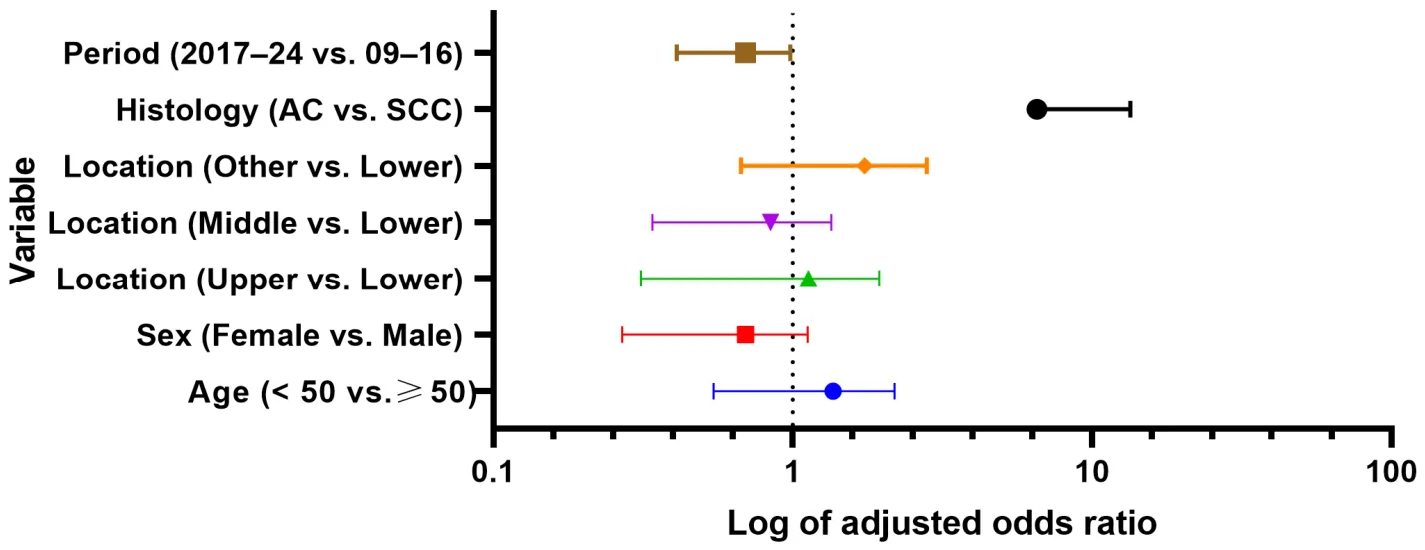
Forest plot of factors associated with advanced-stage esophageal cancer. Adjusted odds ratios (aORs) and 95% confidence intervals (CIs) from the multivariable logistic regression model are displayed on a logarithmic scale. The dashed vertical line at an odds ratio of 1.0 indicates no association. Adenocarcinoma histology was independently associated with higher odds of advanced-stage presentation (aOR 4.16, 95% CI 1.21–14.30; *p* = 0.024), while diagnosis during 2017–2024 showed a non-significant trend toward lower odds of advanced-stage presentation (aOR 0.66, 95% CI 0.43–1.00; *p* = 0.052). Other variables were not statistically significant. AC, adenocarcinoma; SCC, squamous cell carcinoma.

**Table 1:** Baseline clinicopathological characteristics and clinical management of esophageal cancer patients at Siriraj Hospital, stratified by stage at diagnosis.

Characteristics	Overall (n = 591)	Early Stage (I–II) (n = 133)	Advanced Stage (III–IV) (n = 458)	*p*-Value
**Age at diagnosis (years)**				
Median (IQR)	61.6 (54.0–70.0)	61.4 (55.0–69.0)	61.7 (54.0–70.5)	0.742
**Age Group**				0.482
<50 years	75 (12.7%)	14 (10.5%)	61 (13.3%)	
≥50 years	516 (87.3%)	119 (89.5%)	397 (86.7%)	
**Sex**				0.22
Male	534 (90.4%)	116 (87.2%)	418 (91.3%)	
Female	57 (9.6%)	17 (12.8%)	40 (8.7%)	
**Anatomical Location**				**0.002**
Upper third/Cervical	69 (11.7%)	17 (12.8%)	52 (11.4%)	
Middle third	208 (35.2%)	64 (48.1%)	144 (31.4%)	
Lower third/GEJ	92 (15.6%)	18 (13.5%)	74 (16.2%)	
Other/Overlapping	222 (37.6%)	34 (25.6%)	188 (41.0%)	
**Histological Type**				**0.011**
Squamous Cell Carcinoma	540 (91.4%)	130 (97.7%)	410 (89.5%)	
Adenocarcinoma	44 (7.4%)	3 (2.3%)	41 (9.0%)	
Others (Undiff./Small cell)	7 (1.2%)	0 (0.0%)	7 (1.5%)	
**Diagnosis Period**				**0.027**
2009–2016	247 (41.8%)	44 (33.1%)	203 (44.3%)	
2017–2024	344 (58.2%)	89 (66.9%)	255 (55.7%)	
**Clinical Management**				
Surgical Resection	106 (17.9%)	30 (22.6%)	76 (16.6%)	0.147
Chemotherapy	306 (51.8%)	81 (60.9%)	225 (49.1%)	**0.022**
Radiotherapy	294 (49.7%)	93 (69.9%)	201 (43.9%)	**<0.001**

Abbreviations: IQR, interquartile range; GEJ, gastroesophageal junction. Notes: Continuous variables are presented as median (IQR) and compared using the Mann–Whitney U test. Categorical variables are presented as numbers (percentages) and compared using the chi-square test or Fisher’s exact test, as appropriate.

**Table 2:** Univariable and multivariable logistic regression analysis of factors associated with advanced-stage esophageal cancer.

Variables	Unadjusted OR (95% CI)	*p*-Value	Adjusted OR (95% CI)	*p*-Value
**Age Group**				
≥50 years	1 [Reference]		1 [Reference]	
<50 years	1.30 (0.70–2.42)	0.399	1.20 (0.64–2.26)	0.577
**Sex**				
Male	1 [Reference]		1 [Reference]	
Female	0.66 (0.36–1.21)	0.184	0.61 (0.32–1.16)	0.131
**Anatomical Location**				
Lower third/GEJ	1 [Reference]		1 [Reference]	
Upper third/Cervical	0.72 (0.34–1.52)	0.384	0.93 (0.43–2.03)	0.861
Middle third	0.54 (0.30–0.98)	**0.044**	0.74 (0.40–1.39)	0.353
Other/Overlapping	1.32 (0.70–2.48)	0.394	1.52 (0.80–2.90)	0.203
**Histological Type**				
Squamous cell carcinoma	1 [Reference]		1 [Reference]	
Adenocarcinoma	4.33 (1.32–14.23)	**0.016**	4.16 (1.21–14.30)	**0.024**
Others (Undiff./Small cell)	N/A	–	–	–
**Diagnosis Period**				
2009–2016	1 [Reference]		1 [Reference]	
2017–2024	0.61 (0.41–0.92)	**0.019**	0.66 (0.43–1.00)	0.052

Abbreviations: OR, odds ratio; CI, confidence interval; GEJ, gastroesophageal junction. Notes: Multivariable logistic regression was performed to identify factors associated with advanced-stage presentation. Variables were selected based on clinical relevance and a trend toward association in univariable analysis (*p* < 0.20). Adjusted odds ratios (ORs) with 95% confidence intervals (CIs) are reported. A two-sided *p*-value < 0.05 was considered statistically significant. N/A: Odds ratio was not estimated because no early-stage cases were observed in the “Others” category.

## Data Availability

The data supporting the findings of this study are derived from the Siriraj Cancer Registry, Siriraj Hospital, Mahidol University. Patient-level data are not publicly available because of institutional policies and the Thai Personal Data Protection Act (PDPA). De-identified data supporting the findings of this study may be made available from the corresponding author upon reasonable request and subject to approval by the Siriraj Institutional Review Board.

## Supplementary Materials

The following supporting information can be downloaded at https://www.mdpi.com/article/10.3390/mps9050138/s1, Table S1: Baseline comparison of included vs. excluded patients.

## Abbreviations

The following abbreviations are used in this manuscript:

AC	Adenocarcinoma
AJCC	American Joint Committee on Cancer
CI	Confidence Interval
GEJ	Gastroesophageal Junction
GERD	Gastroesophageal Reflux Disease
ICD-O-3	International Classification of Diseases for Oncology, Third Edition
IQR	Interquartile Range
OR	Odds Ratio
SCC	Squamous Cell Carcinoma
