# Supplementary material for: A Registry-Informed Framework for Earlier Detection of Esophageal Adenocarcinoma in Thailand: Evidence from a 16-Year Cohort Study
Source: Methods Protoc. 2026 Sep 21;9(5):138. doi: 10.3390/mps9050138 (PMC13707702; doi:10.3390/mps9050138)
Supplement: Supplementary file 1 [file mps-09-00138-s001.zip › mps-4460255-supplementary.pdf]

Table S1: Baseline Comparison of Included vs. Excluded Patients

Characteristic	Included (n = 591)	Excluded (n = 860)	P-value
**Mean Age at Diagnosis (years)**	61.5	62.9	0.081
**Sex (Male, %)**	90.40%	89.10%	0.941
**Histological Subtype**			0.215
SCC	91.40%	86.00%	
Adenocarcinoma	7.40%	7.00%	
**Diagnosis Period (2017-2024, %)**	**58.20%**	**39.50%**	**< 0.001**

Abbreviations: SCC, squamous cell carcinoma.

Notes: Continuous variables are presented as mean values and compared using the independent samples t-test. Categorical variables are presented as percentages and compared using the Chi-square test.
